# Urolithin Metabotypes Can Determine the Modulation of Gut Microbiota in Healthy Individuals by Tracking Walnuts Consumption over Three Days

**DOI:** 10.3390/nu11102483

**Published:** 2019-10-16

**Authors:** Izaskun García-Mantrana, Marta Calatayud, María Romo-Vaquero, Juan Carlos Espín, María V. Selma, María Carmen Collado

**Affiliations:** 1 Department of Biotechnology, Institute of Agrochemistry and Food Technology, IATA-CSIC, 46980 Valencia, Spain; 2 Department of Biotechnology, Center for Microbial Ecology and Technology (CMET), Ghent University, 9000 Gent, Belgium; marta.calatayudarroyo@ugent.be; 3 Laboratory of Food & Health, Research Group on Quality, Safety and Bioactivity of Plant Foods, CEBAS-CSIC, 30100 Murcia, Spain; mrvaquero@cebas.csic.es (M.R.-V.); jcespin@cebas.csic.es (J.C.E.); mvselma@cebas.csic.es (M.V.S.)

**Keywords:** walnuts, polyphenol, urolithins, metabotypes, gut microbiota, *Gordonibacter*, personalised nutrition

## Abstract

Walnuts are rich in polyphenols ellagitannins, modulate gut microbiota (GM), and exert health benefits after long-term consumption. The metabolism of ellagitannins to urolithins via GM depends on urolithin metabotypes (UM-A, -B, or -0), which have been reported to predict host responsiveness to a polyphenol-rich intervention. This study aims to assess whether UMs were associated with differential GM modulation after short-term walnut consumption. In this study, 27 healthy individuals consumed 33 g of peeled raw walnuts over three days. GM profiling was determined using 16S rRNA illumina sequencing and specific real-time quantitative polymerase chain reactions (qPCRs), as well as microbial activity using short-chain fatty acids analysis in stool samples. UMs stratification of volunteers was assessed using ultra performance liquid chromatography–electro spray ionization–quadrupole time of flight–mass spectrometry (UPLC-ESI-QTOF-MS) analysis of urolithins in urine samples. The gut microbiota associated with UM-B was more sensitive to the walnut intervention. *Blautia, Bifidobacterium*, and members of the Coriobacteriaceae family, including *Gordonibacter*, increased exclusively in UM-B subjects, while some members of the Lachnospiraceae family decreased in UM-A individuals. *Coprococcus* and *Collinsella* increased in both UMs and higher acetate and propionate production resulted after walnuts intake. Our results show that walnuts consumption after only three days modulates GM in a urolithin metabotype-depending manner and increases the production of short-chain fatty acids (SCFA).

## 1. Introduction

Nuts are a significant source of macronutrients (e.g., proteins, unsaturated fats, polysaccharides, fiber), micronutrients (e.g., vitamins and minerals), and bioactive compounds (e.g., tocopherols, phytosterols, phenolic compounds [1]). Ellagitannins (ETs) and ellagic acid (EA) are polyphenols present in nuts, pomegranates, and berries [2]. The human gut microbiota can metabolize ETs and EA into the bioactive metabolites urolithins by lactone-ring cleavage, decarboxylation, and dehydroxylation reactions [3]. Epidemiologic studies have associated long-term nut consumption with a reduced incidence of coronary heart disease, hypertension, and cholesterol-lowering effect [4]. The health-related effects of nut consumption may be linked to gut microbiota, which can release bioactive compounds with higher bioavailability and functionality than parental compounds [5]. Several studies have demonstrated changes in gut microbial communities after the intake of ETs-containing foods on week timescale [2,6,7,8,9,10]. Besides, some of these studies revealed a personalized effect of foods containing ETs [6], which supports the concept of personalized nutrition. According to how ETs metabolize via gut microbiota, individuals can be stratified into three urolithin metabotypes (UMs) [11,12]. Metabotype A (UM-A) is characterized by the production of urolithin-A (Uro-A). Metabotype B (UM-B) is characterized by the production of urolithin-B (Uro-B), isourolithin-A (IsoUro-A), and Uro-A. Metabotype 0 (UM-0) does not produce these final urolithins [11]. Recently, three urolithin-producing bacteria belonging to the genus *Gordonibacter* (*G. pamelaeae* and *G. urolithinfaciens*) and *Ellagibacter isourolithinifaciens* were isolated from human feces [13,14,15,16]. UMs have been proposed as potential cardiovascular disease (CVD) risk biomarkers [17]. Furthermore, UMs were associated with the host blood lipid profile and with the interindividual variability in the improvement of cardiovascular risk biomarkers after the consumption of pomegranate ellagitannins for six weeks [6,17]. In this scenario, this study aims to evaluate the impact of a short-term (three days) nutritional intervention with walnuts on the gut microbiota composition and function in healthy individuals according to their UMs.

## 2. Materials and Methods

### 2.1. Study Design and Participants

A total of 27 healthy individuals (Valencia, Spain) were enrolled in the study. The inclusion criteria were for participants to be older than 18 years old, have a non-declared pathology, and not have taken antibiotics, medication, or pre/probiotics in the two months before the study. The intervention consisted of an intake of 33 g of walnuts per day for three days. A total of 100 g of peeled walnuts provided with 65 g of fats (SFA: 6.4 g; MUFA: 10 g; PUFA: 48 g), 4 g of carbohydrates, 15 g of protein, 12 g of dietary fiber, and 0.02 g of salt. Proanthocyanidins were quantified after acid-catalysis in the presence of phloroglucinol, as previously reported [18]. The analysis of hydrolysable tannins was performed after an acid hydrolysis of nut samples using a method previously reported [19]. 

Walnut samples contained (mg/g fresh weight; mean ± SD): ellagic acid 4.15 ± 0.60; gallic acid 0.48 ± 0.07; and catechin 0.44 ± 0.04. At the beginning (T0) and the end (T3) of these time-points, participants collected fecal samples for gut microbiota analysis and short fatty acids (SCFA) determination and urine for urolithin determination and subsequent UMs stratification. Moreover, each participant filled out a questionnaire about their clinical, anthropometric, and nutritional records at the end of the interventional study. Additionally, the intake of ellagitannins-containing food, as pomegranate and berries were restricted throughout the intervention study. BMI was calculated and stratified as following: lean–normal weight (≤25.0 kg/m^2^), overweight (25.0–30.0 kg/m^2^), and obese (≥30.0 kg/m^2^) [20]. Written informed consent was obtained from all the volunteers. The local ethics committee of Atención Primaria-Generalitat Valenciana (CEIC-APCV) approved the study protocol. All experiments were carried out following approved guidelines and regulations.

### 2.2. Dietary Estimation

The dietary intake of the participants was determined through a comprehensive 140-item validated food frequency questionnaire (FFQ) [21]. In all cases, we validated the FFQ information registered by participants with a three-day food record questionnaire for the intake of dietary nutrients. FFQ records were transformed to energy, macro, and micronutrients daily intake using the nutrient food composition tables developed by the Centro de Enseñanza Superior de Nutrición Humana y Dietética (CESNID) and analyzed by EASY DIET software. Moreover, a 14-item, PREDIMED (PREvención con DIeta MEDiterránea) validated test was used to appraise adherence of participants to the Mediterranean diet [22].

### 2.3. Biological Samples

Each volunteer collected urine and fecal samples at home using plastic containers following standardized protocol given by the team. Then, those were placed in the freezer at −20 °C overnight, before sending to the laboratory where the samples were stored at −80 °C until analysis.

### 2.4. Urolithin Quantification in Urine

Urolithins were identified in urine samples after the three-day nut intervention using ultra performance liquid chromatography–electro spray ionization–quadrupole time of flight–mass spectrometry (UPLC-ESI-QTOF-MS), as previously described [23]. The individuals’ stratification, according to their UMs (UM-A, UM-B, and UM-0), was carried out as previously described [24].

### 2.5. Gut Microbiota Composition Analysis

DNA was extracted from fecal samples using a commercial kit, the Master-Pure DNA Extraction Kit (Epicentre, Madison, WI, USA), following the manufacturer’s instructions with modifications described in García-Mantrana [25]. Purification of the DNA was performed using the DNA Purification Kit (Macherey-Nagel, Duren, Germany) according to the manufacturer’s instructions. 

Specific primers (F_Gordon and R_Gordon) and a TaqMan probe (P_Gordon) targeting the 16S rRNA gene of *Gordonibacter* genus were used for quantification via the real-time quantitative polymerase chain reactions (qPCR) [26], because *Gordonibacter* genus appears in a very low relative abundance, and is not possible to detect by next-generation sequencing (NGS) methods. A touch-down qPCR protocol was applied for DNA amplification and the ramping profile was: 1 cycle at 95 °C for 5 min, followed by the first 20 cycles of 94 °C for 30 s, an auto-increment of 0.5 °C from 55 to 65 °C for 45 s, 72 °C for 2 min. Then, the second 20 cycles of 94 °C for 30 s, 55 °C for 45 s, and 72 °C for 2 min were applied. Finally, it was added 1 cycle at 72 °C for 5 min.

Gut microbiota composition and diversity were determined by V3-V4 variable region of the 16S rRNA gene sequencing, following Illumina protocols. After 16S rDNA gene amplification, Nextera XT Index Kit (Illumina, San Diego, CA, USA) was used for the multiplexing step and a Bioanalyzer DNA 1000 chip (Agilent Technologies, Palo Alto, CA, USA) for checking the PCR product quality. Libraries were sequenced using a 2 × 300 pb paired-end run (MiSeq Reagent kit v3) on a MiSeq-Illumina platform (FISABIO sequencing service, Valencia, Spain), according to the manufacturer’s instructions (Illumina). Statistical analyses of the 16S rRNA gene sequence data were performed with QIIME statistical tools [27]. Chimeric sequences and sequences that could not be aligned were removed from the data set. Sequences that were classified as Cyanobacteria and Chloroplasts were also removed.

### 2.6. Statistical Analysis

Calypso software version 8.84 was used with total sum normalization (TSS) for data mining, multivariate testing, including a redundancy analysis (RDA) and parametric tests [28]. Subsequently, beta diversity based on the Bray Curtis distance (non-phylogenetic) was carried out. To test significance between groups, PERMANOVA test was used. Alpha diversity indexes (Chao1, Simpson, and Shannon) were also determined. Linear discriminant analysis effect size (LEfSe) was used to detect bacterial features between metabotypes and conditions. Data were classified by metadata factors. Graphpad Prism v. 5.04 was used for *t*-test analysis according to data normality assessed by Kolmogorov-Smirnov and Shapiro-Wilk test. Pearson correlations between relative abundances of bacterial groups and SCFA levels were done using Calypso software. Data were considered statistically significant at *p* < 0.05.

### 2.7. Gut Microbiota Activity Analysis

Fecal supernatants were obtained from 100 mg of fecal sample in 1 mL of buffered saline solution. Short Chain Fatty Acids (SCFA) analysis was carried out in the fecal supernatants that were filtered through 0.45 µm-pore-size nitrocellulose filters (Millipore, Burlington, MA, USA). C2-C8 fatty acids analysis was performed as described [29].

## 3. Results

### 3.1. Characteristics of Subjects

The analysis of urolithins in urine after three days of walnut consumption was used for clustering the individuals according to their UMs: UM-A (*n* = 14), UM-B (*n* = 13), and UM-0 (*n* = 0). No significant differences were found in the demographic, clinical, and nutritional characteristics, as well as the Mediterranean diet score according to UMs (Table 1).

### 3.2. Baseline Microbiota Composition is Urolithin-Metabotype Dependent

**Figure 1:**
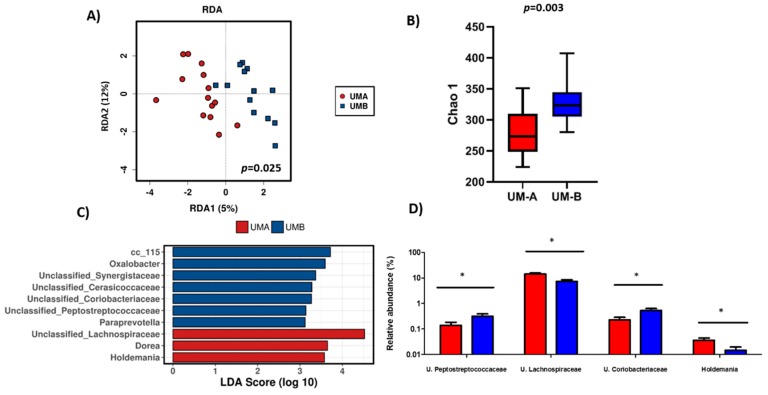
Gut microbiota composition and diversity according to metabotypes (UM-A and UM-B) before the intervention (T0). (**A**) Redundancy analysis (RDA) on the genus level. (**B**) Gut microbial richness was measured by Chao index. (**C**) Linear discriminant analysis (LDA) effect size (LEfSe) plot of taxonomic biomarkers identified in the gut microbiome of volunteers. (**D**) Relative abundances on genus level in UM-A and UM-B. ANOVA test was used to test significance differences in the relative abundances according to metabotype. Results are presented as mean ± SD. Significant differences (*p* < 0.05) are marked with an asterisk (*).

Baseline microbiota (T0, *n* = 27) was characterized by the presence of Firmicutes with a mean relative abundance and SD of 77.31% ± 2.88, followed by Bacteroidetes (15.86% ± 0.28), Actinobacteria (3.13% ± 0.65), Verrucomicrobia (1.78% ± 1.22), and Proteobacteria (1.31% ± 1.50) at the phylum level. The relative abundance of phylum Synergistetes was significantly enriched in UM-B (*p* < 0.05) compared to UM-A. At the family level, the family Lachnospiraceae was associated with UM-A, whereas the abundance of the families Cerasicoccaceae, Peptostreptococcaceae, Synergistaceae, and Paraprevottelaceae were related to UM-B. A redundancy analysis (RDA) on genus level showed significant differences in the microbial population, according to UMs (*p* = 0.025) (Figure 1A). Volunteers belonging to UM-B had a higher richness (*p* = 0.003, Chao 1 index) than UM-A (Figure 1B).

Lefse analysis showed that *Dorea*, *Holdemania* genus, and members of Lachnospiraceae family were enriched in UM-A, while genus cc_115, *Oxalobacter*, members of Synergistaceae, Cerasicoccaceae, Coriobacteriacea, Peptostreptococcaceae family, and *Paraprevotella* were associated with UM-B individuals (Figure 1C).

### 3.3. Microbiota Composition of UM-B is Sensitive to Walnuts Consumption

**Figure 2:**
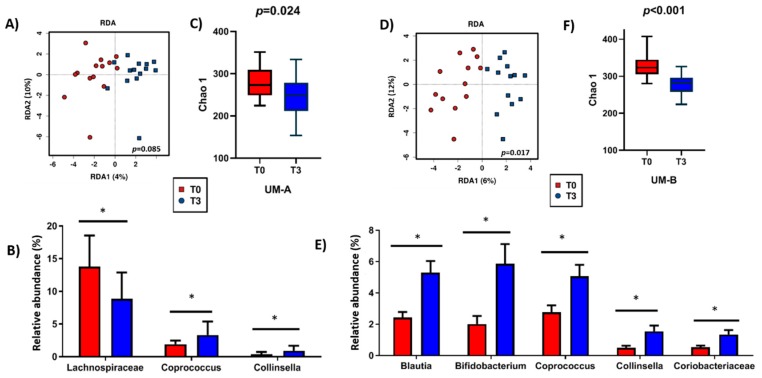
Effect of the intervention with walnuts in gut microbiota composition (**A**,**B**) and diversity (**C**) in UM-A and in gut microbiota composition (**D**,**E**) and diversity (**F**) in UM-B. ANOVA test was used to test significance differences in the relative abundances before and after the intervention. Alpha diversity measured by Chao1 index. Results are presented as mean ± SD. Significant differences (*p* < 0.05) are marked with an asterisk (*).

The redundancy analysis (RDA) of all individuals (*n* = 27) showed that bacterial communities were significantly affected by the three day walnut intervention (*p* = 0.001). However, when the volunteers were stratified according to their UMs, these shifts were only significant for UM-B (*p* = 0.017) (Figure 2D). At the phylum level, walnut intake decreased the relative abundance of Bacteroidetes and increased Actinobacteria when all individuals were considered (*p* < 0.05). These differences were consistent for UM-B after clustering. At the family level, walnut consumption increased the Coriobacteriaceae family in both UMs, while the Bifidobacteriaceae family increased after three days only for UM-B volunteers. Among bacterial genera, members of the Lachnospiraceae family decreased after the walnut intervention in UM-A individuals, *Coprococcus* and *Collinsella* increased in both UMs, whereas *Blautia, Bifidobacterium,* and unclassified Coriobacteriaceae increased only in participants belonging to UM-B (*p* < 0.05) (Figure 2B,E). According to the Chao1 index, gut microbial richness significantly decreased for UM-A (*p* = 0.024) and UM-B (*p* < 0.001) after walnut consumption (Figure 2C,F).

### 3.4. Walnut Consumption Increased Faecal Gordonibacter Levels in UM-B

**Figure 3:**
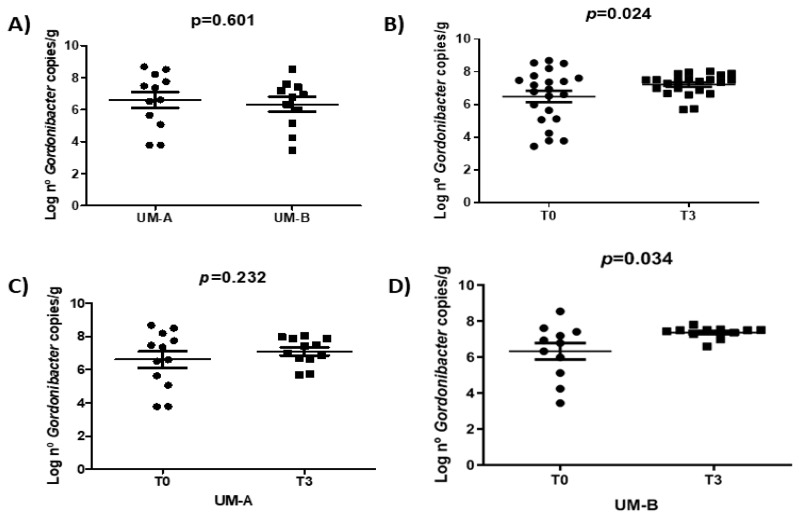
Microbiota composition before (T0) and after the intervention (T3). *Gordonibacter* levels measured using qPCR according to UMs before the intervention (**A**). *Gordonibacter* levels before and after the intervention for all the participants (**B**). *Gordonibacter* levels before and after the intervention for UM-A (**C**) and UM-B participants (**D**). Paired t-test was used to test significance differences in *Gordonibacter* levels before and after the intervention. Results are presented as mean ± SEM.

Before the intervention, UM-A tended to present higher counts of *Gordonibacter*. However, it was not significant compared to UM-B (*p* = 0.601) (Figure 3A). After intervening and analyzing the individuals as a single group, a significant increase of fecal *Gordonibacter* levels was observed (*p* = 0.024) (Figure 3B). After clustering, according to UMs, walnut consumption only increased *Gordonibacter* levels in UM-B individuals (*p* = 0.034) (Figure 3C,D). No significant associations with BMI, MD stratification, nutrient intake, or sex with *Gordonibacter* levels were found.

### 3.5. Effect of Walnut Consumption on Gut Microbiota Activity

**Figure 4:**
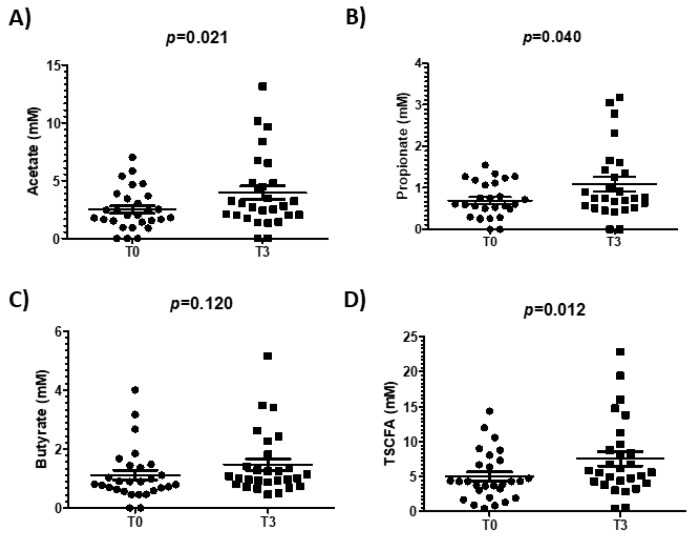
SCFA profile before and after the intervention for all the participants. Acetate (**A**), propionate (**B**), butyrate (**C**) and total SCFA (**D**) levels measured by GC for all the participants. A paired t-test was used to test significant differences in SCFA levels before and after the intervention. Results are presented as mean ± SEM.

Before the intervention, volunteers belonging to UM-A tended to present higher levels of butyrate (*p* = 0.070). After the intervention, we did not find significant differences in SCFA production after walnut consumption according to UMs (data not shown), but analyzing all participants as a single group, the walnut intake increased the total SCFA (*p* = 0.012) levels, specifically acetate (*p*= 0.021) and propionate (*p* = 0.040) (Figure 4).

### 3.6. Associations between Gut Microbiota Composition/Activity and Walnut Intervention

Before the walnut intervention, at the genus level a positive correlation was found between the increase in acetate and *Phascolarctobacterium* (*p* = 0.006) and *Anaerostipes* (*p* = 0.011), propionate and *Phascolarctobacterium* (*p* = 0.040), *Coprococcus* (*p* = 0.031) and *Anaerostipes* (*p* = 0.011), and butyrate and *Coprococus* (*p* = 0.030) and *Anaerostipes* (*p* = 0.020), respectively. At the species level, we found that this positive correlation between propionate and butyrate with *Coprococus* was due to *Coprococcus catus*. After the walnut intervention, positive correlations were observed between acetate levels and *Phascolarctobacterium* (*p* = 0.018) and *Dehalobacterium* (*p* = 0.014), propionate levels and *Eubacterium* (*p* = 0.024), *Phascolarctobacterium* (*p* = 0.040), *Slackia* (*p* = 0.024), and butyrate levels with *Dehalobacterium* (*p* = 0.016), and *Eubacterium* (*p* = 0.006).

## 4. Discussion and Conclusions

Understanding individuals’ response to dietary bioactive compounds such as polyphenols is essential in the context of personalized nutrition [30]. The occurrence of specific gut microbiota metabotypes can affect the metabolism and bioactivity of polyphenols and the response of individuals upon polyphenol consumption can vary [6]. Our present study shows that the impact exerted by the three day walnut intervention on the gut microbiota depends on the urolithin metabotypes (UMs) of healthy individuals. Therefore, our results add significance to previous studies that aimed to explain the controversial benefits of bioactive compounds present in foods due to the inherent high inter-individual variability [6]. Notably, only UM-B participants responded significantly to the intervention with walnuts towards a change in the gut microbiota composition. 

Parameters such as baseline gut microbiota composition and habitual daily intakes may influence a differential host response [31]. Furthermore, gut microbiota composition would be considered as a predictor for the inter-individual variability in response to dietary interventions [31]. Although UM groups did not present differences in their dietary intakes, significant differences in the gut microbial composition, according to UMs clustering, were observed. In agreement with another study [32], higher relative abundances of Synergistetes phylum and members of Coriobacteriaceae family were observed in UM-B individuals, whereas members of the Lachnospiraceae family predominated in UM-A. We also found a higher gut microbiota richness in UM-B individuals in agreement with Romo-Vaquero and colleagues [32]. Differences of basal microbiota in UMs may be linked to the individual response to ETs intake. It has been reported that intervention with an ellagitannin-rich pomegranate extract for three weeks exerted a differential impact on cardiovascular risk markers of individuals depending on their UM metabotype [6]. The cardiovascular benefits were mainly observed in UM-B individuals, who initially presented a higher cardiovascular risk [6]. These results are in agreement with our present study, where only UM-B subjects responded significantly to the walnut intervention. Moreover, a previous study that evaluated the capacity to produce nut phenolic metabolites in subjects with metabolic syndrome showed that several urolithins significantly increased after a nut-enriched-diet. However, this pathology is associated with an altered gut bacterial diversity [33].

Overall, the evidence on the impact of walnuts on the gut microbiota is scarce and mainly provided by long-term interventions where the significance of the gut microbiota metabotypes, linked to the inter-individual variability, was not explored [8,9,34]. In agreement with González-Sarrías et al., our results supported the personalized effect associated with ellagitannin-containing foods consumption [6]. In the present study, a three day walnut intervention modulated some microbial groups, including the increase of *Blautia, Bifidobacterium* and bacterial members of Coriobacteriaceae, such as *Gordonibacter*, but only in UM-B individuals. Previous research has shown that walnut consumption increased the relative abundance of butyrate-producing species, although the authors observed opposite results on some bacterial groups such as *Ruminococcus* and Bifidobacteria [8,35]. Holscher et al. assessed the impact of consuming 42 g walnuts per day for two three week periods on the human gut microbiota composition and observed a decrease in the relative abundance of *Ruminococcus* and Bifidobacteria [8]. However, another recent study showed that the same amount of walnuts per day enhanced the abundance of Ruminococcaceae and Bifidobacteria [35]. The effect of walnut consumption on the gut microbial composition may be linked to ellagic acid and ellagitannins, but other polyphenols and other compounds such as dietary fiber and unsaturated fats [4] would also be influencing factors.

The highest content in omega-3 fatty acids characterizes walnuts with respect to other nuts [36,37]. Although the impact of polyunsaturated fatty acids on the gut microbiota is poorly defined, several human studies have shown some common changes in the gut microbiota after omega-3 PUFA supplementation. In particular, a decrease in *Faecalibacterium*, from Ruminococcaceae family, often associated with an increase in other butyrate-producing bacteria belonging to the Lachnospiraceae family has been observed [38]. Walnuts present similarities in the chemical composition with other nuts, like almonds and pistachios, which do not contain ellagitannins. Some studies have investigated the effects of other dried fruits and nuts on the gut microbiota composition [9,34,39]. Ukhanova et al. demonstrated enrichment in butyrate producers after almonds and pistachios consumption for 18 days [34]. Holscher and colleagues performed an interventional study with almonds and found an increase in the relative abundances of several butyrate-producing bacteria such as *Lachnospira, Roseburia,* and other bacterial groups such as *Dialister* and *Oscillospira* [9]. In any case, all these studies focused on changes in gut microbial composition and agreed on the same point that these interventions produced an increase in butyrate-producing bacteria. In our study, a three-day walnut intervention was enough to increase acetic acid and propionate of fecal samples, without differences between UMs. Riviére et al. reported health benefits of propionate in mice by reducing serum cholesterol, improving insulin sensitivity, and promoting satiety [40]. Recently, the use of inulin-propionate ester, to deliver propionate to the colon selectively, improved insulin resistance in overweight and obese subjects [41].

A recent study (including 365 study participants in 13 trials) found that diets enriched with walnuts led to lower total and LDL-cholesterol when compared with other diets [42]. It has also been reported that walnut consumption is associated with a lower risk of type 2 diabetes [4]. The PREDIMED trial is the largest primary prevention trial showing that an intervention to promote a Mediterranean diet, which is beneficial against the incidence of several major chronic diseases in subjects at high cardiovascular risk, particularly when improved adherence to the Mediterranean diet includes increased consumption of extra virgin olive oil and mixed tree nuts. The PREDIMED trial showed that participants included in the MD group supplemented with nuts presented a lower risk of cardiovascular events and type 2 diabetes [43,44].

The increase in acetate production is in accordance with the increase in the relative abundances of the *Bifidobacterium* genus. Moreover, acetate is used by cross-feeding species as a co-substrate to produce butyrate [40]. Therefore, this could mean that the increase of *Bifidobacterium* could favor the colonization of other beneficial butyrate-producing bacteria, such as *Coprococcus*, leading to an increase in butyrate production. Although we did not observe a significant increase in butyrate production, which could have been due to the short-term walnut intervention, we found positive correlations between higher acetate and propionate production, and *Phascolarbacterium, Coprococcus*, and *Anaerostipes*. These results agree with previous studies that described the production of SCFA, including acetate and propionate, by *Phascolarctobacterium* [45] as well as the production of butyrate by *Coprococus* and *Anaerostipes* [40]. At the species level, we also observed positive correlations between *Coprococcus catus*, butyrate, and propionate concentrations. This is related to the capability of this bacterial species for butyrate and propionate production, via butyryl-CoA: acetate CoA-transferase route and acrylate pathway, respectively [45]. It is well known that butyrate exerts health benefits, including the preservation of the gut barrier integrity. Remarkably, it has been demonstrated that urolithin-A (Uro-A), as a microbial metabolite, significantly enhances the gut barrier function through the up-regulation of the epithelial tight junctions’ proteins [46], which confirmed pioneering investigations of our group that demonstrated for the first time the preservation of the colon architecture by Uro-A under intestinal inflammation [2]. Although fecal SCFA profiles are widely used as markers of gut health or disease, caution on interpreting the results may be required. More than 95% of SCFAs in the gut are quickly absorbed. Therefore, measurement of fecal SCFAs concentration only represents the 5% of produced SCFA left unabsorbed. Luminal concentrations of SCFAs can be affected by both rates of production and disappearance, circadian rhythms, section of the intestine, and specific microenvironment at luminal or mucosal compartments [47].

In summary, the main findings of this interventional study show that walnut consumption for three days is enough to modulate gut microbial composition in a UM-depending manner and to increase the production of SCFA. This study supports the concept of personalized nutrition and that not all the dietary recommendations may exert the same benefits in all individuals. This concept is growing and there is enough evidence that explains that a complex relationship between diet and microbiome could be behind this. Nevertheless, there are still difficulties predicting gut microbiota and host responses to a given dietary intervention. There is still no consensus because there are many parameters that we should take into account, such as host physiology, habitual dietary patterns, and gut microbiota baseline. Therefore, more research is needed and clinical trials should take into account this inter-individual variability.

## Figures and Tables

**Table 1:** Clinical and nutritional characteristics of the participants.

		Urolithin Metabotypes		
	Total (*n* = 27)	UM-A (*n* = 14)	UM-B (*n* = 13)	*p* Value
Age (years)	39.5 ± 7.3	36.1 ± 7.2	43.1 ± 5.6	0.521
BMI (kg/cm^2^)	23.3 ± 3.2	23.9 ± 3.5	22.7 ± 2.8	0.625
Normal Weight (%)	70.4	64.3	76.9	0.961
Overweight (%)	29.6	35.7	23.1	0.393
Sex				
Female (%)	55.5	57.1	61.5	0.471
Male (%)	40.7	42.8	38.5	0.565
MD (%)	55.5	57.1	53.8	0.669
Total Protein (g/day)	93.9 ± 15.5	95.5 ± 13.5	92.2 ± 17.9	0.597
Animal-Derived Protein (g/day)	47.7 ± 13.9	50.8 ± 12.4	44.4 ± 15.1	0.237
Plant-Derived Protein (g/day)	44.1 ± 7.7	42.4 ± 7.4	45.9 ± 7.9	0.232
Lipids (g/day)	78.8 ± 9.5	80.9 ± 8.6	76.5 ± 10.3	0.246
SFA	16.5 ± 3.2	16.1 ± 3.5	16.9 ± 3.0	0.527
MUFA	33.5 ± 4.9	33.7 ± 5.4	33.3 ± 4.5	0.844
PUFA	12.8 ± 2.5	12.6 ± 2.1	13.0 ± 2.9	0.684
Total Carbohydrates (g/day)	215.4 ± 29.7	207.8 ± 24.3	223.6 ± 33.6	0.171
Dietary Fiber (g/day)	26.1 ± 5.9	26.7 ± 6.4	25.4 ± 5.7	0.593
Insoluble Dietary Fiber (g/day)	16.8 ± 4.8	17.2 ± 4.9	16.3 ± 4.8	0.636
Soluble Dietary Fiber (g/day)	3.1 ± 0.8	3.0 ± 0.9	3.2 ± 0.8	0.553

Results are presented as mean ± SD and percentage (%). SFA: saturated fatty acids; MUFA: monounsaturated fatty acids; PUFA: polyunsaturated fatty acids. *p* < 0.05 for comparison the clinical and nutritional characteristics between UMs. MD: Mediterranean diet adherence.

## Abbreviations

GM	gut microbiota
UM	urolithin metabotype
qPCR	real-time quantitative polymerase chain reaction
UHPLC/Q-TOF-MS	ultra-high performance liquid chromatography-quadrupole time-of-flight mass spectrometry
ETs	ellagitannins
EA	ellagic acid
Uro	urolithin
IsoUro	isourolithin
CVD	cardiovascular disease
SCFA	short chain fatty acids
BMI	body mass index
FFQ	food frequency questionnaire
CESNID	Centro de Enseñanza Superior de Nutrición Humana y Dietética
PREDIMED	PREvención con DIeta MEDiterránea
LEfSe	linear discriminant analysis effect size
RDA	redundancy analysis
